# Morphological, anatomical and histological studies on knob and beak characters of six goose breeds from China

**DOI:** 10.3389/fphys.2023.1241216

**Published:** 2023-08-28

**Authors:** Yang Zhang, Xinlei Xu, Wangyang Ji, Shangzong Qi, Qiang Bao, Yong Zhang, Yu Zhang, Qi Xu, Guohong Chen

**Affiliations:** ^1^ Key Laboratory for Evaluation and Utilization of Poultry Genetic Resources of Ministry of Agriculture and Rural Affairs, Yangzhou University, Yangzhou, China; ^2^ Yangzhou Tiangge Goose Industry Development Company Limited, Yangzhou, China

**Keywords:** goose, knob, morphological, histological, beak

## Abstract

The knob serves as both a sexual indicator of a goose’s maturity and a significant packaging attribute that garners consumer attention. However, studies regarding the morphological, anatomical and histological traits of different breeds and ages on the on knob in goose are lacking. In this study, six breeds with typical goose knob types were selected, and their knob size, morphological, anatomical and histological traits were characterized. The results showed that: Knob was more prominent in gander than in female goose, and the difference was the most obvious in Magang goose. Wanxi white goose and Shitou goose had the largest knob bulge, while Magang goose and Sichuan white goose were smaller. The total knob volume of Wanxi White goose and Shitou goose was significantly higher than that of other breeds, regardless of male or female (*p* < 0.05). The beak volume of Wanxi White goose and gander was significantly higher than that of other goose breeds (*p* < 0.05). Furthermore, the observation revealed that the “knob” primarily consisted of skin-derived tissue and bony protrusions. As age advances, the knob of both male and female geese undergoes synchronous development, with the knob of male geese typically surpassing that of their female counterparts during the same period. The growth rate of knob in male goose was the fastest from 70 to 120 days of age, and slowed down from 300 to 500 days of age. The growth rate of knob in female goose was slower than that in male goose. There were essential differences in the composition of Yangzhou goose knob and Magang goose knob. The subcutaneous tissue of Magang goose was rich, and the thickness of epidermis, dermis and various layers was significantly smaller than that of Yangzhou goose (*p* < 0.05). With the growth of goose knob, the cells of the epidermal spinous layer became denser and gradually condensed into an overall structure, and there was a clear boundary between the dermis and epidermis after adult. In adulthood, the fiber fascicle network was staggered and dense, with greater toughness and elasticity, and the stratum corneum, epidermis, reticular layer, dermis and other skin structural layers became thicker.

## Introduction

Packaging traits play an important role in poultry breeding, mainly including feather color, shin color, and carcass fullness (Eitan et al., 1998). Packaging traits can directly remind consumers of the quality of poultry products and affect their purchase desire. For example, consumers often judge the health status of chickens through the lodging/upright characteristics of chicken combs (Stettenheim, 2000; Mukhtar and Khan, 2012). Different regions and customs are also directly mapped to the packaging of poultry (Zhang et al., 2020). For example, the color of chicken feathers includes yellow feathers and white feathers (Wang et al., 2022). White is a symbol of purity in Europe, and Europeans’ preference for white makes white feathers more popular. In summary, packaging traits and their genetic rules in poultry have long been valued by poultry breeders (Tufvesson et al., 1999; Li et al., 2022).

The packaging characters of geese mainly include feather color, shin color and so on (Hamadani et al., 2014). For example, Yangzhou geese with 3 Gy spots are favored by consumers and occupy a dominant position in the consumer market (Yan et al., 2019). The knob is also a type of consumption preference, which it is possible to directly determine the male and female geese as well as the age of the goose (Deng et al., 2022). It is a typical packaging trait in the sales of live geese and processed meat products of goose head (Deng et al., 2021).

Packing characters such as feather color and shin color have been studied in various local geese (Ren et al., 2021; Xu et al., 2022), but there is no detailed report on goose knob. The protrusion of the head is the most common in gallinales birds, and its formation is mainly concentrated in the skull. In contrast, bony prominences on the skull are less common in other clades, while prominences in certain areas of the beak are more common (Breeze et al., 2021). The cassowary (*Casuarius spp*.) has a bony bulge on the dorsal side of the skull, formed mainly by the mesethmoid bone, while the guinea fowl (*Numida meleagris*) has a bony helmet from the frontal bone (Stettenheim, 2000). In Anseriformes, bony prominences were formed by the expansion of the anterior orbitals and/or foreheads. In some Galliformes, bony prominences are associated with their covering of signaling epidermal structures, so bony prominences may be associated with some physiological, auditory, or sensory functions (Stettenheim, 2000).

As a sex modification, the bony bulge is linked to an individual’s health, immunity and ability to reproduce. For example, the mute swan’s knob can change with the time of year and age, and females have larger knob than usual during the breeding season (Horrocks et al., 2009).

At the same time, head bumps are associated with various ecological adaptations, including muscle attachment points, thermal regulation, acoustic regulation, species identification, defense and aggression. For example, the outer bony bulge of guinea fowl is composed of keratin, and the whole is pyramidal, with large differences between male and female individuals, which can be used for intraspecific identification and protection of the group from predators (Angst et al., 2020). The cassowary’s bony bulge may resemble a resonance chamber for low-frequency communication, and its size and shape are intrinsically related to the vocal signal. Until now, the bird skull growth map is still being studied, and the bony bulge is more likely to evolve in land or waterfowl, where the bulge makes the head more exposed. As a marker, bony prominences play an irreplaceable role in intrasecific information transmission (Stettenheim, 2000).

The variety resources of geese are mainly distributed in Europe, Caucasus and Asia, which can be roughly classified into European geese and Chinese geese (Moniem et al., 2020). China has rich genetic resources for geese, according to statistics, China has nearly 90% of domestic geese livestock population in the world, and is one of the countries with the largest number of geese and the richest breed resources in the world (China Committee on Animal Genetic Resources, 2011; Zhang et al., 2019). There are 30 local goose breeds in China, all of which are descended from *Anser cygnoides* (*Anser cygnoides*) except Yili goose (Yang et al., 2019). China’s local geese can be divided into white goose species (such as Sichuan white goose, Jiangnan white goose) and grey goose species (such as Shitou goose, Magang goose). Compared with European geese, Chinese geese have a slender neck and grow into adults with a fleshy bulge at the top of the bill, known as a knob, which is a distinguishing feature. The skin of the knob is closely connected to the skin of the beak. However, whether the knob is a bulge in the upper beak or a bony bulge in the frontal bone needs further investigation. Although all domestic Chinese geese have a typical knob across the base of the beak and near the forehead, only a smaller bulge was found in the ancestor (*Anser cygnoides*), suggesting that the knob was formed by long-term artificial breeding in Chinese geese. Knob presents incomplete dominant inheritance. Chinese geese cross with European geese, showing varying degrees of variation between the two species. Broadly speaking, knobs tend to be more sizable in males compared to females, and they consistently exhibit greater dimensions in adults than in juveniles. (Lee, 2021). Knob is not only a sign of sexual maturity of geese, but also one of the important packaging traits concerned by consumers. Consumers have obvious consumption preference for large knob geese. Therefore, it is of certain practical value to carry out research on knob formation for expanding the consumption of goose meat. The size of knob mainly depends on species and age, but what are the differences in histology and anatomy of knob of different species and different age, and what is the internal mechanism of the size difference? This series of problems has not been reported so far.

To understand the factors affecting the size of knob is the basis for breeding and improving the size of knob. In this study, Six goose breeds with typical knob types, such as Lion-head goose, Magang goose, Wanxi white goose, Zhedong goose, Yangzhou goose, (All were 500 days of age) and Sichuan goose, were selected. Additionally, the sizes of different breeds and morphological traits of knob were investigated. Furthermore, the histological of knob were determined. These data will reveal the growth and divergences of knob between different breeds, and provide a reference for revealing the molecular mechanism of knob trait formation and promoting genetic improvement of goose knob trait. To further meet people’s consumption preferences and expand meat goose consumption.

## Materials and methods

### Animals and sample collection

All animal experiments were approved by with the Institutional Animal Care and Use Committee of Yangzhou University (approval number: 151–2018). Six breeds of Shitou goose, Magang goose, Wanxi white goose, Zhedong white goose, Yangzhou goose, Sichuan white goose were provided by the National Waterfowl Resource Bank (Taizhou) for the measurement and analysis of knob and beak size. Each breed was 60, with half male and half female. Approximately 500 healthy male Yangzhou geese were raised at Yangzhou Tiange Goose Industry Co., Ltd (Yangzhou, China). according to the farm’s standard practice. These geese were used to determine knob size at 70, 120, 300 and 500 days of age. When reaching the specified age, six geese with large knob or small knob were selected, respectively, and then the birds were humanely euthanized (electronarcosis followed by bleeding). The apical integumentary outgrowth (including skin and subcutaneous connective tissue) tissue of the knob was collected, fixed in 4% paraformaldehyde and taken for paraffin sections. The remaining parts were labeled, stored with sealed in −20°C refrigerator and then used for skull taxidermy.

### Determination of knob morphological index

In order to determine knob and beak size, we have established a set of methods for the determination of knob and beak in mute swan in China (Horrocks, Perrins and Charmantier, 2009), which has been published (Ji, et al., 2021). The length, height, and width of the knob were measured in millimeters. The knob length (Kl) was measured between the most anterior part of the knob and the phenotypic boundary of knob. The knob width (Kw) was measured at the widest part of the knob. The knob height (Kh) was measured between the fronto-nasal junction and the most dorsal part of the knob. Knob size was assessed by the product of knob length, width and height.

### Determination of knob histological index

Knob morphological traits were determined using Hematoxylin and Eosin Stainning (Ji, et al., 2021). The skin samples were fixed in 4% paraformaldehyde for 24 h. The samples were placed in an embedding cassette and rinsed with running water (to remove the fixative from the tissue) for 30 min, and the samples were dehydrated in a graded ethanol series. The paraffin blocks were cut (Leica Biosystems, Wetzlar, Germany) along the horizontal axis into 3 μm-thick sections and stained with hematoxylin and eosin (HE) according to standard protocols. The samples were scanned using a NanoZoomer scanner (Hamamatsu, Sydney, Australia). The fiber diameter and cross-sectional area were calculated using an image analysis system (Image-Pro Plus, Media Cybernetics, Rockville, MD, United States).

### Preparation of skull specimen and determination of knob size

**FIGURE 1:**
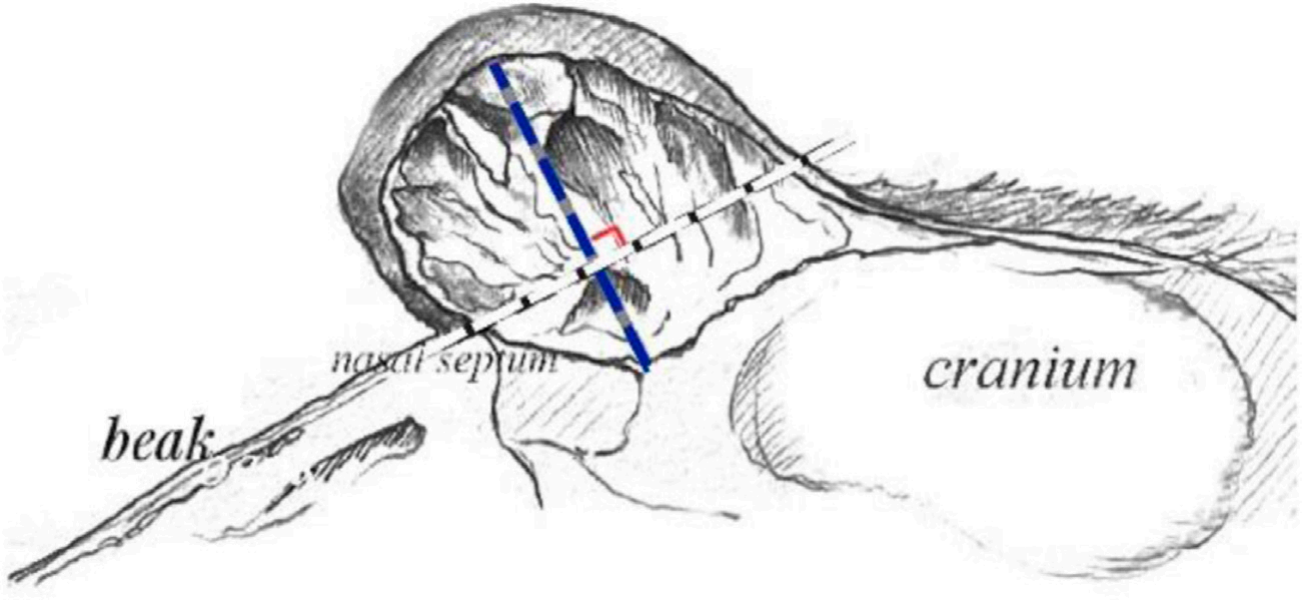
Schematic diagram of measurement of bony crest. Note: The length of the bony crest is the maximum depth from the outer wall of the bone to the bottom of the hollow chamber, the bony crest depth line is perpendicular to the beak plane line. The blue line is to depict the length of the bony crest, and the white line is to make the bony crest depth clearer.

Head feathers, skin and most of the muscles were removed by natural degradation. The processed goose heads were placed in plastic bags and sealed for 3 months (summer), and the corresponding labels were written. After the muscle tissue was decomposed, the goose heads were removed and washed with tap water. Remove impurities as much as possible, wash and dry. If the oil returns after drying, it can be soaked in acetone for 2 weeks and then taken out to dry. This method can minimize the damage of chemicals to bones and help to preserve the basic structure and surface structural characteristics of bones (Zhao et al., 2000). The measurement procedure of bone bulge was according to the measurement method of knob, and the depth of bone bulge is illustrated in Figure 1.

### Statistical analysis

Results are shown as means with corresponding standard deviation (SD). Comparisons of repeat measurements among different breeds and different ages were conducted using SPSS statistical software (version 13.0; SPSS Inc., Chicago, IL, United Sttaes). Duncan’s multiple range test was used to analyze the main effects among different breeds. Statistical significance was set at *p* ≤ 0.05.

## Results

### Comparison of morphological differences of knob and beak among different adult goose breeds

**FIGURE 2:**
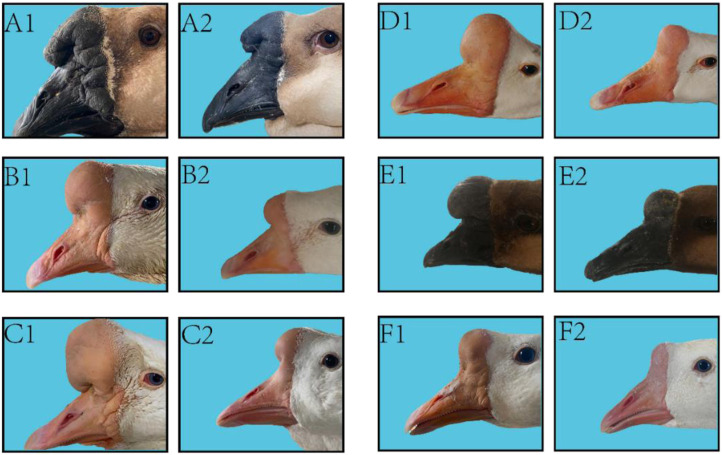
Morphological observation on knob and beak of 6 adult geese. Note: 6 types of geese with typical knob size were selected for photographing and phenotypic observation, **A1**: Shitou goose ♂; **A2**: Shitou goose ♀; **B1**: Zhedong white goose ♂; **B2**: Zhedong white goose ♀; **C1** Wanxi White Goose ♂; **C2**: Wanxi White Goose ♀; **D1**: Yangzhou goose ♂; **D2**: Yangzhou goose ♀; **E1**: Sichuan white goose♂; **E2**: Sichuan white goose; **F1**: Magang goose ♂; **F2**: Magang goose ♀.

The knob of male and female geese of six breeds including Shitou goose, Zhedong geese, Wanxi white geese, Yangzhou geese, Sichuan geese and Magang geese were tested at the age of 500 days. The specific phenotypes are shown in Figure 2. Combined with Table 1, it can be seen that among all breeds, the knob was significantly more prominent in male geese than in female geese, and the difference was the most obvious in Magang geese. The Wanxi white goose and Shitou goose had the largest knob protrusion, while Magang goose and Sichuan goose had smaller knob. The total volume of the Wanxi white goose and Lion head geese was significantly higher than that of the other geese, regardless of male or female (*p* < 0.05), and the Wanxi white goose was larger than Shitou goose (*p* < 0.05). The difference in knob volume was mainly reflected in the length, width and height of the knob of different breeds.

**TABLE 1:** 6 kinds of male geese knob and beak sizes comparison.

Breed	Sex	Knob length/mm	Knob width/mm	Knob height/mm	Knob volume/cm^3^	Beak length/mm	Beak width/mm	Beak height/mm	Beak volume/cm^3^
Shitou goose	♂	38.97 ± 7.01^ab^	46.44 ± 5.90^a^	39.90 ± 4.63^b^	73.79 ± 5.83^b^	67.81 ± 2.98^c^	40.02 ± 1.99^a^	39.17 ± 2.56^a^	106.62 ± 3.31^ab^
	♀	31.97 ± 4.34^a^	38.13 ± 1.92^b^	33.33 ± 5.59^b^	41.55 ± 3.47^b^	68.81 ± 3.96^c^	37.89 ± 1.76^a^	37.75 ± 3.18^a^	98.93 ± 3.86^a^
Zhedong white goose	♂	33.85 ± 5.09^bc^	39.75 ± 4.10^b^	36.57 ± 4.1^bc^	50.61 ± 4.07^c^	76.35 ± 4.36^b^	37.29 ± 1.81^b^	34.83 ± 2.78^bc^	99.98 ± 4.44^bc^
	♀	26.31 ± 4.74^b^	34.36 ± 2.57^c^	30.15 ± 4.96^bc^	28.23 ± 3.20^c^	72.17 ± 4.04^b^	34.15 ± 1.84^b^	32.04 ± 2.58^c^	79.64 ± 3.62^b^
Wanxi white goose	♂	42.8 ± 7.41^a^	47.19 ± 7.46^a^	48.27 ± 7.10^a^	102.07 ± 10.89^a^	80.73 ± 3.02^a^	37.73 ± 1.81^b^	36.34 ± 3.08^b^	111.13 ± 4.02^a^
	♀	33.58 ± 6.99^a^	40.83 ± 6.61^a^	40.01 ± 6.14^a^	57.91 ± 7.40^a^	77.18 ± 2.46^a^	35.21 ± 1.91^b^	34.85 ± 1.55^b^	94.86 ± 2.37^a^
Yangzhou goose	♂	32.68 ± 8.78^d^	36.09 ± 5.92^b^	34.3 ± 8.51^c^	46.13 ± 10.24^c^	77.58 ± 3.53^b^	36.47 ± 2.36^b^	35.43 ± 2.36^b^	100.80 ± 4.07^abc^
	♀	27.15 ± 4.52^b^	32.8 ± 2.65^c^	28.31 ± 3.18^c^	26.01 ± 2.31^cd^	75.45 ± 3.46^a^	34.9 ± 2.24^b^	34.7 ± 2.77^b^	91.95 ± 3.70^a^
Sichuan white goose	♂	29.19 ± 3.46^d^	35.63 ± 2.27^b^	29.83 ± 3.16^d^	31.52 ± 2.17^c^	76.94 ± 2.37^b^	36.49 ± 2.14^b^	33.45 ± 1.86^cd^	93.99 ± 2.36^c^
	♀	20.75 ± 3.50^c^	29.43 ± 2.53^d^	22.31 ± 3.55^d^	14.10 ± 1.38^e^	69.79 ± 5.56^bc^	31.89 ± 1.89^c^	29.71 ± 1.65^d^	66.26 ± 2.22^c^
Magang goose	♂	34.32 ± 8.06^bc^	35.89 ± 4.56^b^	29.67 ± 4.06^d^	38.79 ± 4.98^c^	69.88 ± 4.21^c^	33.18 ± 2.35^c^	32.18 ± 2.29^d^	75.04 ± 3.08^d^
	♀	24.27 ± 4.60^c^	28.62 ± 2.70^d^	23.21 ± 2.82^d^	16.69 ± 1.8^de^	67.01 ± 3.65^c^	30.07 ± 1.74^d^	29.35 ± 2.83	59.62 ± 2.80^c^

Note: Each types of geese contains male and female, among which pair-to-pair comparison was made for the same sex, knob volume = knob length × knob width × knob height, beak volume = beak length × beak width × beak height, and different lowercase letters on shoulder marks indicated significant differences (*p* < 0.05).

For beak type, there were small differences in beak size between gander and female geese of all breeds and large differences between breeds. The bill volume of gander was significantly higher than that of other geese species (*p* < 0.05). There was no significant difference in the beak volume between female Wanxi white goose and Yangzhou goose or Lion head geese The differences in beak volume were mainly in the length and width of the beak among different breeds. White geese show a longer beak in appearance, and gray geese show a broad, thick, and short beak type in appearance.

### Anatomical differences of knob of different species

**FIGURE 3:**
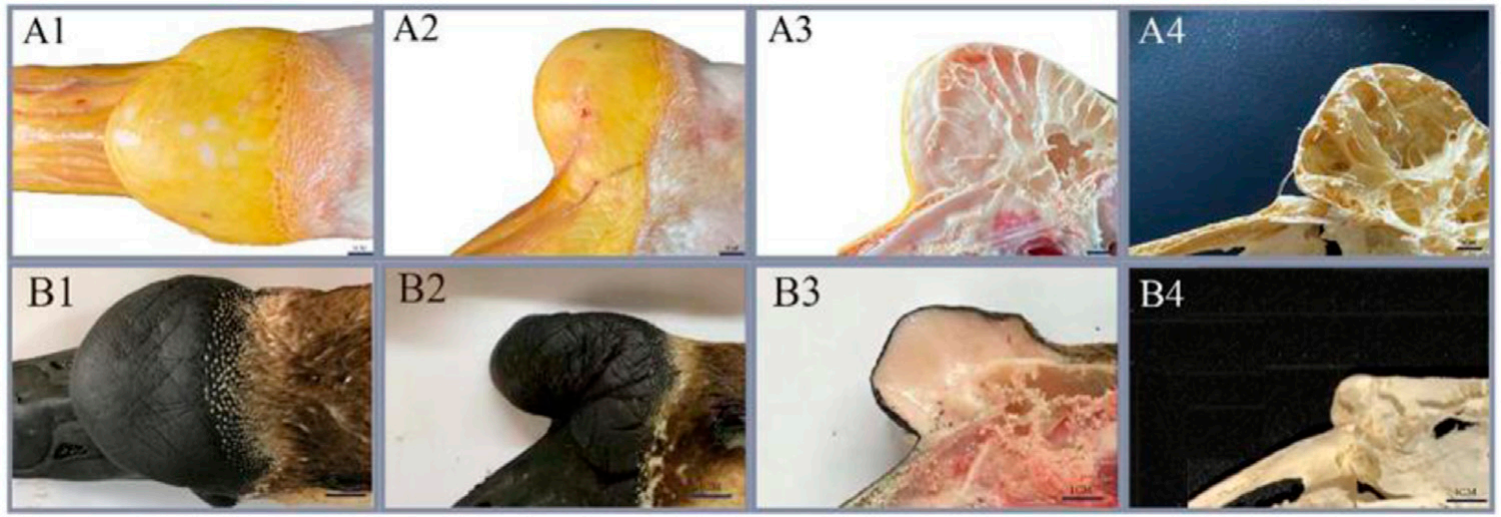
Comparison of phenotype and anatomy in goose knob with different breeds. Note: Fresh head specimens of Yangzhou goose and Magang goose were collected respectively. Morphology of knob was observed; fresh head were prepared by half dissection and skull specimens were made. **A1**: top view of the head of Yangzhou goose, **A2**: side view of the head of Yangzhou goose, **A3**: head profile of Yangzhou goose, **A4**: bone profile of the head of Yangzhou goose. **B1**: top view of the goose head, **B2**: side view of the goose head, **B3**: longitudinal section of the goose head, **B4**: longitudinal section of the goose head skeleton.

The knobs of Yangzhou geese and Magang geese of typical size at 500 days of age were dissected. The results showed that the knobs were mainly composed of skin derived tissues and bony prominences, and the proliferative skin was thicker than normal skin without feather follicles. The morphological differences of knobs of different varieties were as follows: The knob of Magang geese was dark and softer to the touch than that of Yangzhou geese. Anatomical differences among different varieties of knob were as follows: Most of Yangzhou goose knob are bony protrusions. From the longitudinal profile of the bony protrusions, its structure is similar to that of loofah, and the frontal profile is honeycomb. The bony bulge consists of two pairs of vaporized frontal bone on both sides. With the increase of age, the gasification bulge gradually heals into a sphere, and the larger the bony bulge, the thinner the bony bulge wall. The entire skull can be disassembled through the nasofrontal junction with the beak and eventually the globular convex bone, suggesting that the knob is located in the craniofacio-frontal bone rather than part of the beak. Magang goose also has bony protrusion, but it is obviously smaller than Yangzhou goose. Hyperplastic knob skin is mostly composed of adipose tissue mass derivatives (Figure 3).

**FIGURE 4:**
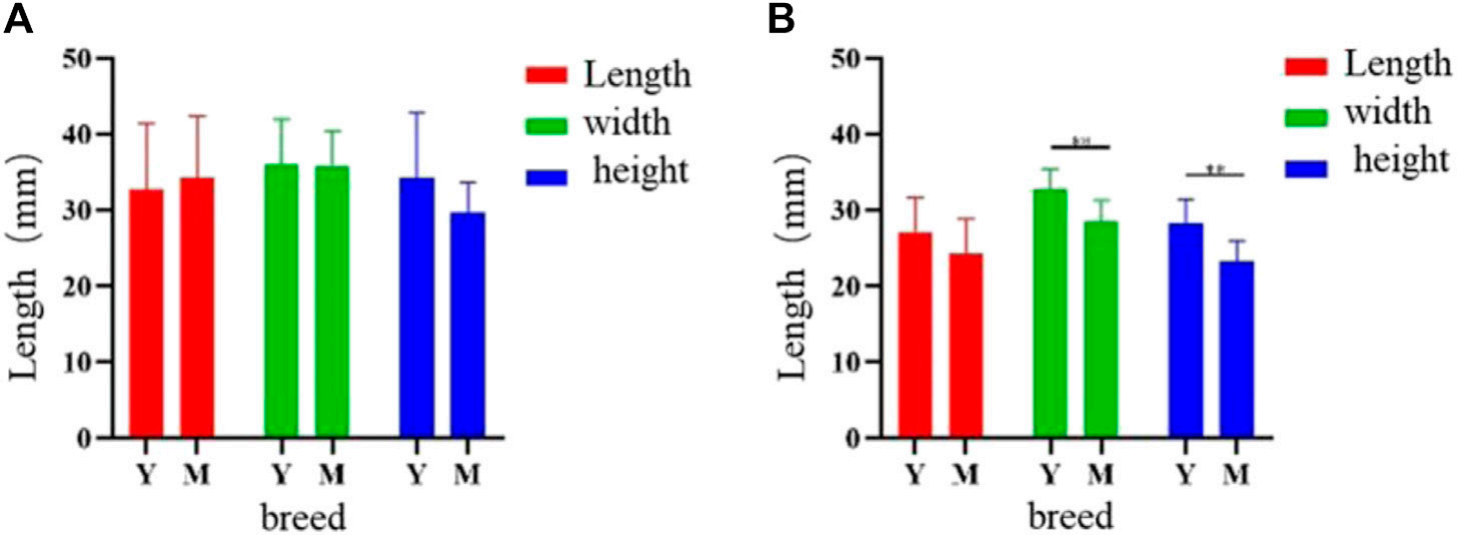
Sizes difference of goose knob and bone crest in different varieties. Note: The length, width and height of knob **(A)** and bony crest **(B)** in Yangzhou goose and Magang goose were measured respectively. *X*-axis represents variety, *Y*-axis represents measurement length, unit: mm, Y: Yangzhou goose M: Magang goose, ** means the difference is extremely significant (*p* < 0.01), * means the difference is significant (*p* < 0.05).

Analysis of the two knob sizes showed that the greater width and height were showed in female Yangzhou geese than Magang geese (*p* < 0.05), And can be higher than 0.7% and 14% respectively. (Figure 4).

### Comparison of morphology and anatomy of knob of different age

**FIGURE 5:**
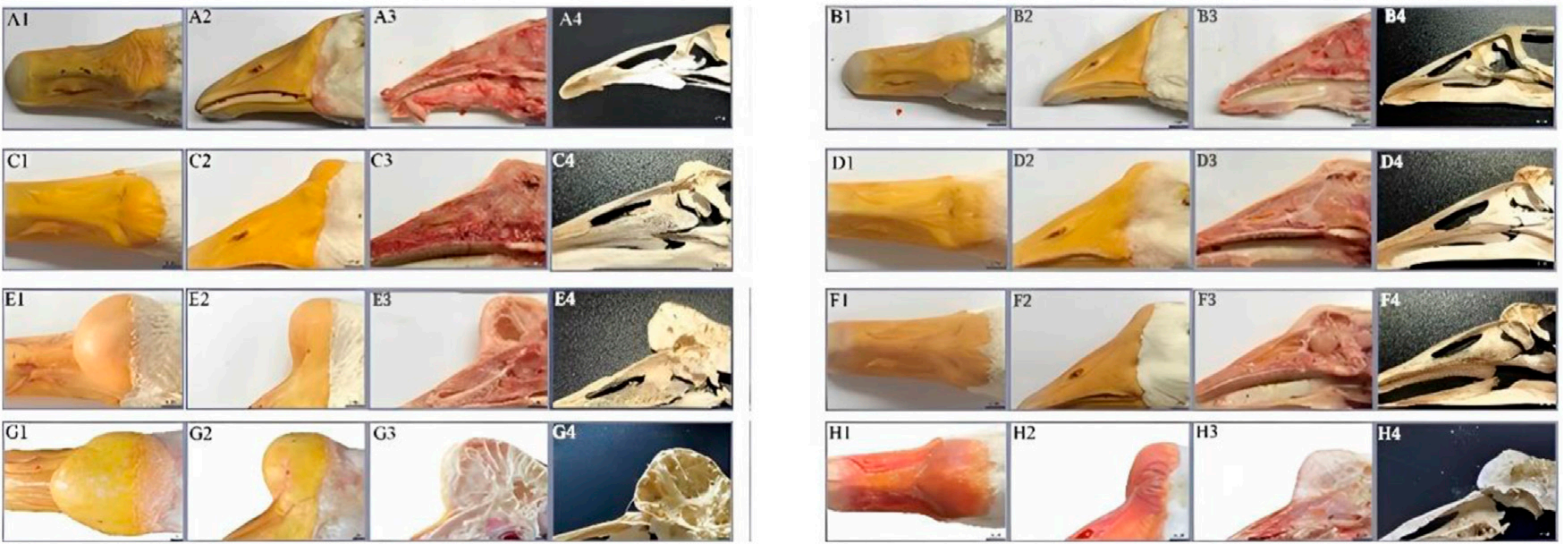
Anatomical comparison of Yangzhou geese at 70, 120, 300 and 500 days of age. Note: Yangzhou geese at the age of 70 days, 120 days, 300 days, and 500 days were collected respectively. Morphology of knobs was observed, and half anatomy and bone specimens were prepared. **A1**: head view of 70-day-old male gander, **A2**: head side of 70-day-old male gander, **A3**: head longitudinal section of 70-day-old male gander, **A4**: Longitudinal section of head skeleton of a 70-day-old gander; **B1**: the head view of the 70-day-old geese, **B2**: the head profile of the 70-day-old geese, **B3**: the head longitudinal profile of the 70-day-old geese, **B4**: the head longitudinal profile of the 70-day-old geese; C1-C4, D1-D4, E1-E4, F1-F4, G1-G4, and H1-H4 are 120-day-old male and female geese, 300-day-old male and female geese, and 500-day-old male and female geese, respectively.

Anatomical comparison of Yangzhou geese at 70 days of age, 120 days of age, 300 days of age and 500 days of age: Because knob had not formed significantly before 120 days of age, only the knob at 70 days of age was used as reference in this study. Through continuous observation of the longitudinal section, the results showed that the knob of male and female geese developed synchronously, and the knob of male geese was generally larger than that of female geese at the same period. The formation of Yangzhou goose knob was about 100 days old (Figure 5). The morphologic changes of knob were as follows: at 70 days of age, the knob developed from a flat forehead with no protrusion to a humdrum protrusion with two fossa at 120 days of age, and formed a spherical protrusion at 300 days of age. At 500 days of age, the knob skin would extend from the forehead to the rear, and from 70 days of age to 500 days of age, the knob skin mainly showed a substantial increase in length and height. The anatomical changes of knob were as follows: from no protuberant at 70 days of age to hard humdrum bony protuberant at 120 days of age, and to globular protuberant at 300 days of age. With the increase of age, the hardness of the bone bulge will gradually become smaller, and the inner wall of the vaporized bone will gradually become thinner and loose in texture, so that it can be easily crushed. The development of skin hyperplasia tissue was thickened with age.

**FIGURE 6:**
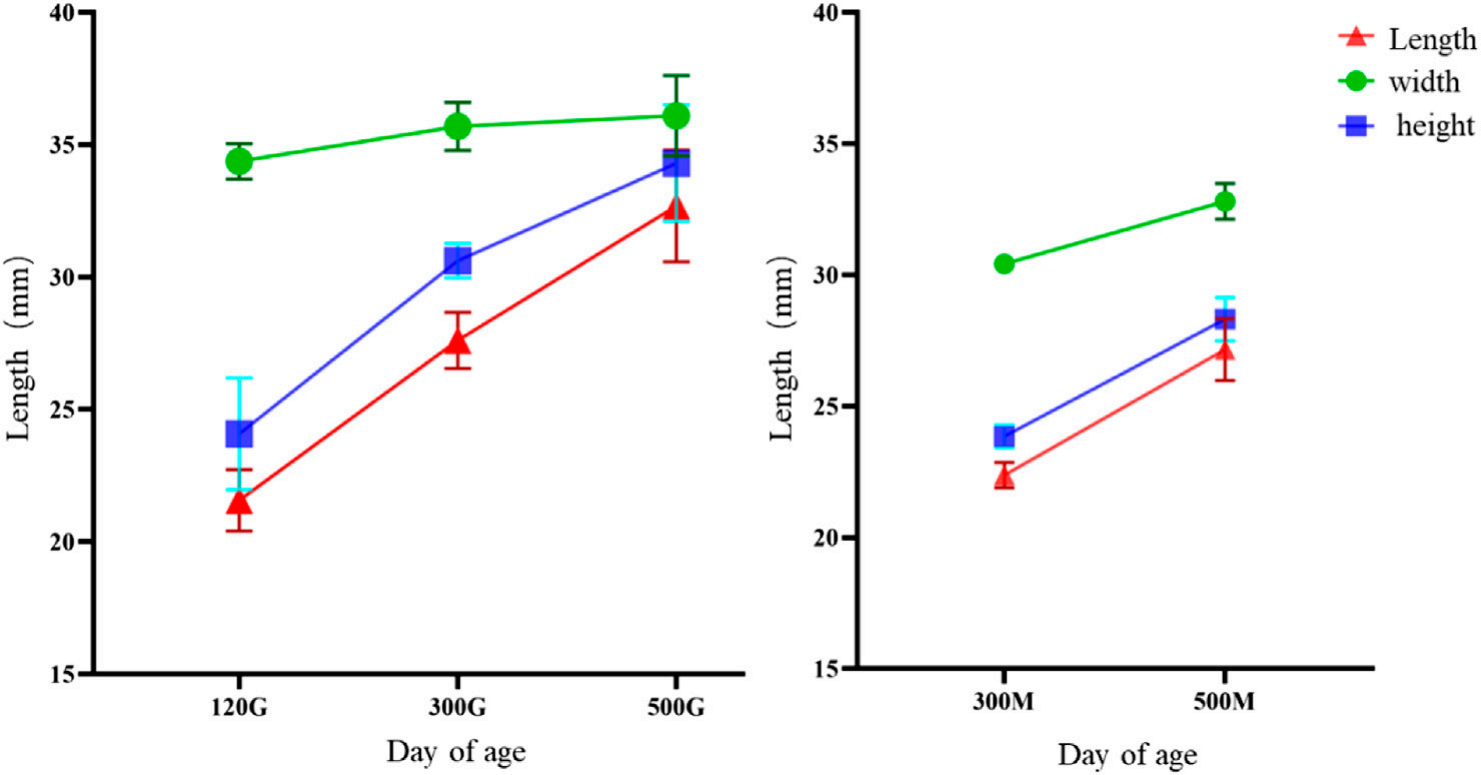
Knob growth curve of different day age. Note: The knob length, width and height of Yangzhou geese at the age of 120 days, 300 days and 500 days were measured respectively; *X*-axis represented the age and gender, and *Y*-axis represented the measurement length, unit: mm; **(A)** male goose; **(B)** female goose.

The size analysis of knob of different days of age showed that the growth rate of knob of geese was the fastest between 70 and 120 days of age, and the growth rate of knob was slower between 300 and 500 days of age. The growth of knob in female geese was slower than in male geese (Figure 6).

### Histological difference of knob of different species and age

**FIGURE 7:**
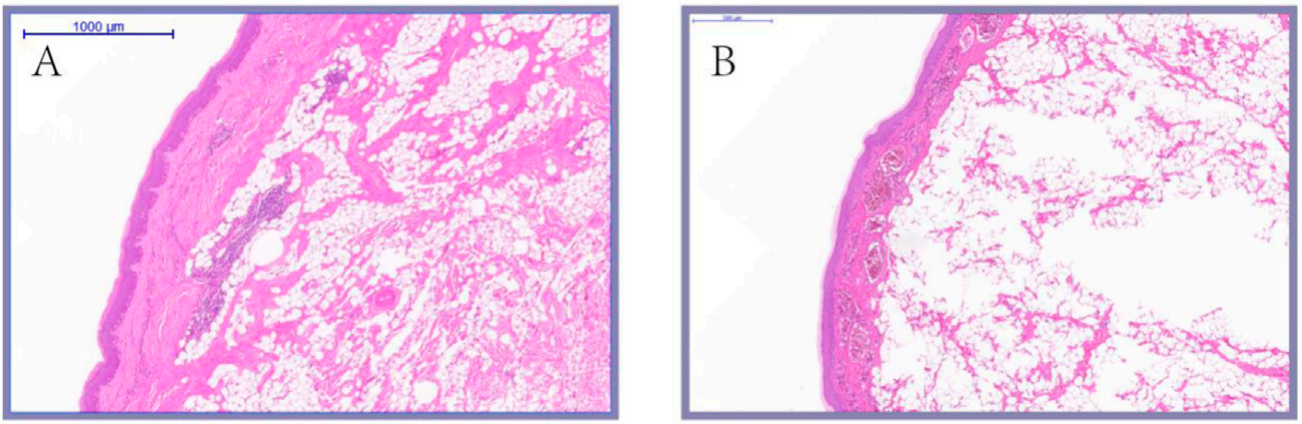
Histological comparison of Yangzhou goose (5×). Note: HE staining was performed on the skin tissues of fixed Yangzhou goose and Magang goose knob. And take pictures with magnification × 5; **(A)** knob skin of Magang goose; **(B)** knob skin of Magang goose.

**FIGURE 8:**
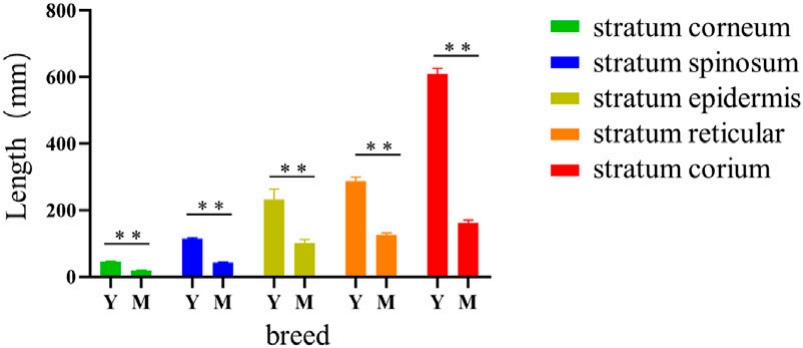
Histological comparison of Yangzhou goose knob skin tissue in different varieties. Note: The area of each layer in skin tissue section was observed and the specific length of each layer was measured; *X*-axis represented the length of tissue in μm; *Y*-axis represented the variety; Y: skin tissue of Yangzhou goose knob; M: skin tissue of Magang goose knob; the stratum corneum, stratum spinosum, stratum epidermis, stratum reticular, and stratum corium were shown in order by side notes; ** indicated extremely significant difference (*p* < 0.01), and * indicated significant difference (*p* < 0.05).

As shown in Figure 7, the histological observation of adult knob of Yangzhou geese and Magang geese showed that there were essential differences in the composition of the knob of Yangzhou geese and Magang geese. The subcutaneous tissue of Magang geese was abundant, and the thickness of epidermis, dermis and various layers were significantly lower than those of Yangzhou geese (*p* < 0.05), And have been correspondingly diminished by approximately 53%, 64%, 57%, 55%, and 73%. (Figure 8).

**FIGURE 9:**
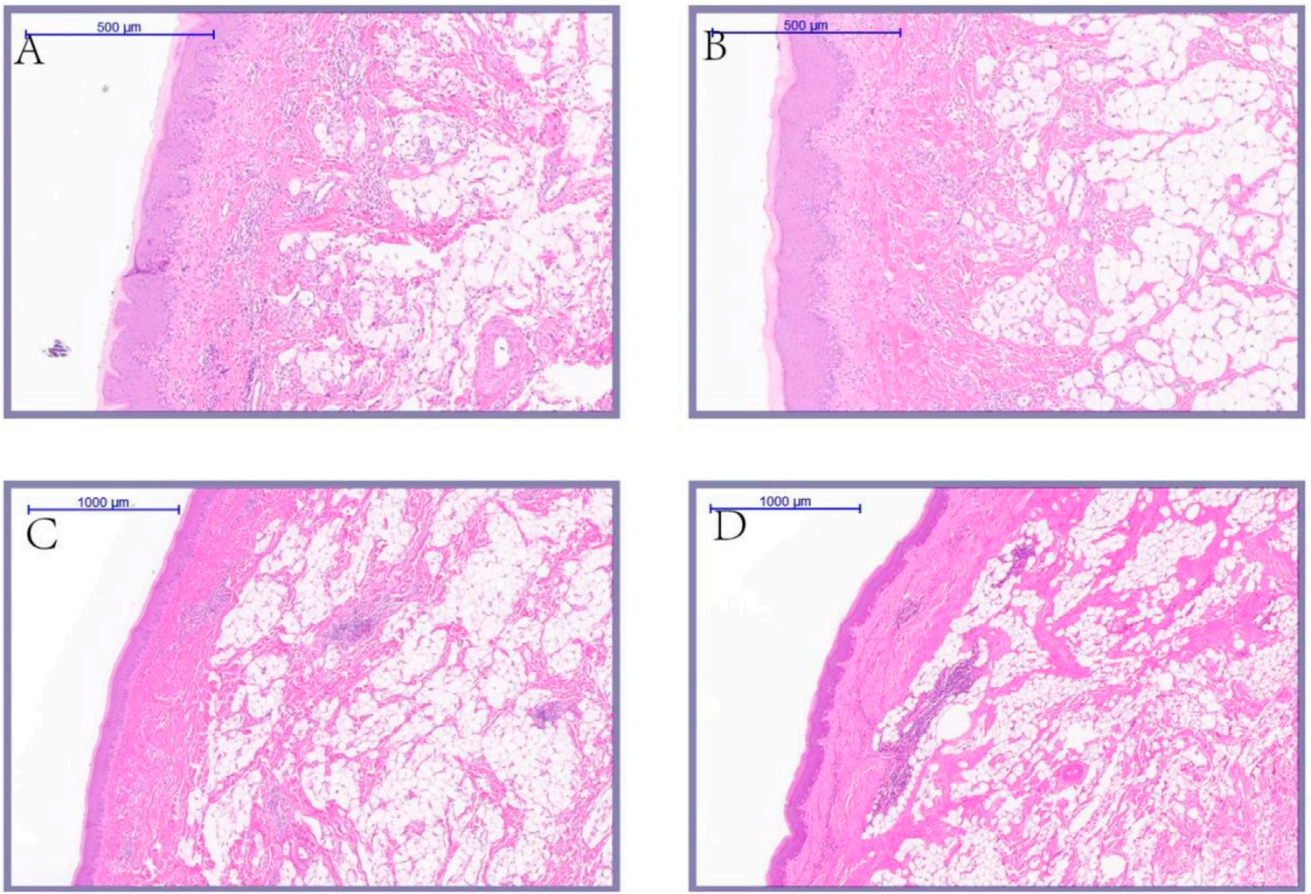
Histological comparison of Yangzhou goose **(A,B)** 7.5×; **(C,D)** 5×). Note: Sampling 70 days of age, 120 days of age, 300 days of age, 500 days of age yangzhou goose knob skin tissue were collected; the knob skin tissue HE staining were made after fixed, and magnification ×5 or 7.5 × pictures; **(A)** knob skin tissue of 70 days age; **(B)** knob skin tissue of 120 days age; **(C)** knob skin tissue of 300 days age; **(D)** knob skin tissue of 500 days age.

**FIGURE 10:**
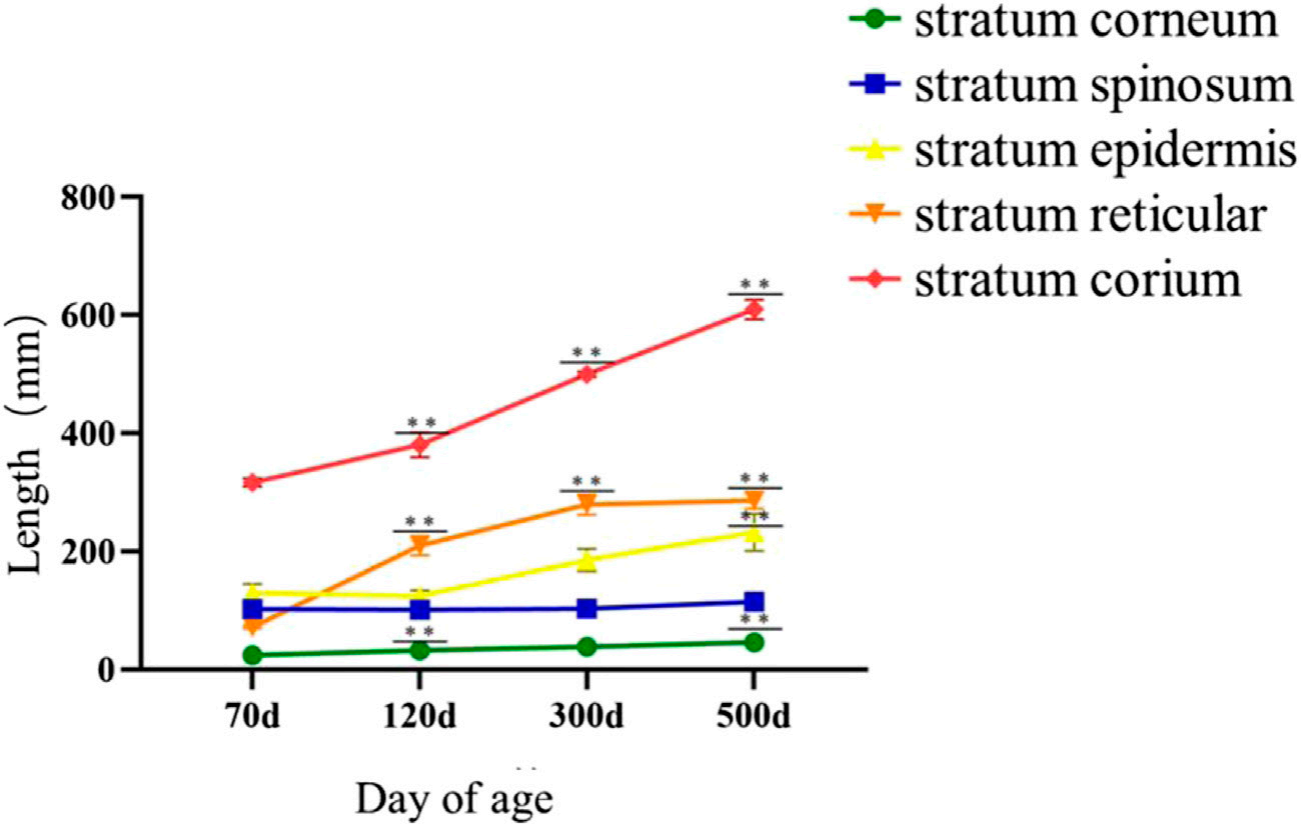
Histological comparison of Yangzhou goose knob at different days of age. Note: The area of each layer in skin tissue of 70-day-old, 120-day-old, 300-day-old and 500-day-old Yangzhou gosknob was observed, and the specific length of each layer was measured. *X*-axis represented tissue length, unit: μm; *Y*-axis represented day age, 70 d: age of 70 days; 120 d: age of 120 days; 300 d: age of 300 days; 500 d: age of 500 days; the stratum corneum, stratum spinosum, stratum epidermis, stratum reticular, and stratum corium were shown in order by side notes; ** indicated extremely significant difference (*p* < 0.01), * indicated significant difference (*p* < 0.05).

As shown in Figure 9, during epidermal development, with the growth of goose knob, the spinous cells become more dense and gradually condense into a large area. The boundary between dermis and epidermis can be clearly seen during the development of goose knob. During dermal development, the connective tissue arrangement becomes dense and the tissue structure is looser at 70 days of age. After development to adulthood, the fiber bundle network becomes interleaved and dense, with greater toughness and elasticity, and each skin structure layer thickens. The overall appearance of the knob is thickened in the stratum corneum, epidermis, reticular layer, and dermis (Figure 10).

## Discussion

The morphology, anatomy and histology of goose knob were observed. The knob consisted of fleshy skin hyperplasia and bony prominences. It has been noticed that knobs cannot be classified strictly as skin derivatives (Mayr, 2018). The outer layer of knobs consists of keratinous skin and fleshy flesh. Instead of a protuberance in the beak, the bulbous bulges are formed by the vaporization and healing of the paired frontal bones above the naso-frontal junction. In both male and female geese, grey goose breeds generally have shorter beaks compared to white goose breeds. Compared with other large geese, Wanxi white geese had a longer beak, and the size of Yangzhou goose’s knob was at the middle level in all the size data, and the beak size accounted for the upper level. Yangzhou geese, as a medium goose breed, had a longer beak and a larger head, which showed potential for knob development and was suitable for breeding (Ji et al., 2021).

In phenotypic and anatomical observations, the skin at the growth site of goose knob at 70 days of age was relaxed, and no thicker skin was formed before 120 days of age. Until adulthood, the skin of knob was much thicker than normal skin tissue. During the development of knob, the bony protrusion expands, which determines the size of knob to some extent. However, the bony protrusion does not seem to determine the size of the knob in Magang goose. For example, the knob in Magang goose is soft in touch and mostly consists of muscle material. Therefore, the relationship between the size of the knob and the bony protrusion in Magang goose needs further study.

Furthermore, notable distinctions in length and height (*p* < 0.05) were observed among Yangzhou geese with varying knob sizes, while no significant variation in width was detected. In the observation experiment, the length and height of the bony bulge were well beyond the range of the frontal bone, but the width of most of the bony bulge coincided with that of the frontal bone. The thickness of the skin within the knob may affect the width of the knob, while the width of the knob or the bone bulge does not completely determine the size of the knob.

A knob or forehead bulge is relatively common in geese birds, such as mute swan and king velvet duck. Knob size, as a secondary sexual trait, is an effective criterion for judging male qualities in all aspects. Studies by Ferns et al. have shown that males with large knobs are better able to resist attacks from other males than those with small knobs (Ferns et al., 2013). Their female partners are able to devote more time to feeding, laying eggs, and hatching, and do not have to run away or alert to attacks from other teat ducks. Most male ducks with very small knobs even run away in pairs to avoid conflict with other male pairs. Except the ducks with the smallest knobs, all of the male ducks with large knobs on their heads made great contributions to reproduction and, accordingly, obtained a corresponding opportunity to reproduce (Ferns et al., 2013). For females, the size of the mute swan knob may be related to its reproductive performance. Nicholas Horrocks et al. found that the mute swan knob during breeding is always larger than that of the mute swan during non-breeding period, except just after laying eggs and hatching (Horrocks et al., 2009). This reflects the physical cost of mute swan breeding and the fact that mute swan knob appears to be a condition-dependent and highly plastic knob trait. Meanwhile, the characteristics of mute swan’s knob character are very similar to that of mute goose. Whether some features of mute swan’s knob character also exist in mute geese deserves further study.

In histological observation, skin tissue also showed significant differences between large and small knobs. During growth and development, the stratum corneum, epidermis, reticular layer, dermis and other tissues show thickening. In geese with large knobs at 500 days of age, the stratum corneum, prickly cell layer, and reticular layer were thicker. A thicker stratum corneum helps protect the skin during knob development. Basal cells are able to continuously proliferate to form spinous layer cells, which are composed of fixed two to three layers of cells and may be established at the beginning of development (Zhongzhi, 2004). However, significant changes have occurred in geese with larger knobs at 500 days of age, possibly due to the more frequent transformation of basal cells into acanthocytes. The reticular layer is located in the dermis and is rich in thick bundles of elastic and collagen fibers (Janson et al., 2017). Similarly, comb is a skin derivative, which is rich in collagen, hyaluronic acid and glycosaminoglycan, mainly derived from the reticular layer and the subcutaneous tissue below the reticular layer (Dang et al., 2011; Driskell et al., 2013). Data from previous studies have shown that the chemical composition of chicken comb has a wide range of industrial uses and great medicinal value (Sida, 2013). The thickness of the reticular layer may directly affect the content of chemical components in the skin tissue, so the thicker the reticular layer was, the higher the content of elastin and collagen was (Chendong, 2018). However, the specific components of knob tissue and its high value of exploitation still need further investigation.

## Conclusion

In this study, we investigated the developmental changes in the sarcomas of the Yangzhou and Magang geese. The morphological variation in the sarcomas of the different species showed that the sarcomas of the Yangzhou goose were orange or orange in colour, while the sarcomas of the Magang goose were darker and softer to the touch than those of the Yangzhou goose. The anatomical variation in the sarcomas of the different species indicates that most of the sarcomas of the Yangzhou goose are bony projections, with the longitudinal section of the bony projections resembling the flesh of a loofah and the frontal section showing a honeycomb structure. These results will provide new insights into the characterisation of sarcomas and provide a theoretical basis for the elucidation of the regulatory mechanisms of packaging traits.

## Data Availability

The raw data supporting the conclusion of this article will be made available by the authors, without undue reservation.
